# The serial mediating effects of moral resilience and moral courage on the relationship between moral injury and positive coping style in Chinese nursing interns

**DOI:** 10.3389/fpsyg.2025.1652127

**Published:** 2025-11-26

**Authors:** Ruixin Zhang, Xue Wang, Jie Chen, Shulin Zhang, Ying Li

**Affiliations:** 1 Department of Orthopaedics, The First Affiliated Hospital of Chongqing Medical University, Chongqing, China; 2 Chongqing Municipal Health Commission Key Laboratory of Musculoskeletal Regeneration and Translational Medicine, The First Affiliated Hospital of Chongqing Medical University, Chongqing, China; 3 Department of Nursing, The First Affiliated Hospital of Chongqing Medical University, Chongqing, China; 4 Orthopedic Laboratory, Chongqing Medical University, Chongqing, China

**Keywords:** coping, mental health, moral courage, moral resilience, moral injury, nursing interns

## Abstract

**Background:**

Clinical practice is a necessary experience for nursing students to become qualified nurses. The impact of moral injury on nursing interns’ coping styles remains unclear. This study aimed to explore the correlation between moral injury and positive coping style among nursing interns in clinical practice and to examine the mediating roles of moral resilience and moral courage on this correlation.

**Methods:**

A convenience sample of nursing interns (*n* = 1,122) from 6 nursing schools in the Southwest China were recruited using social media platforms. The Chinese version of Moral Injury Symptom Scale: Healthcare Professionals Version, Simplified Coping Style Questionnaire, Rushton Moral Resilience Scale and Nurses’ Moral Courage Scale were used to collect data from February 1 to April 15, 2024. Regression-based mediation analysis was used to distinguish the direct effect of moral injury on positive coping style, and the indirect effect mediated by moral resilience and moral courage.

**Results:**

Moral injury was negatively associated with moral resilience (*r* = −0.463, *P* < 0.001), moral courage (*r* = −0.226, *P* < 0.001) and positive coping scores (*r* = −0.235, *P* < 0.001). Moral resilience was positively correlated to moral courage (*r* = 0.184, *P* < 0.001) and positive coping scores (*r* = 0.196, *P* < 0.001). Moral courage was positively associated with positive coping scores (*r* = 0.515, *P* < 0.001). The mediation effect test indicates that moral injury can directly influence positive coping (*p* < 0.01), with the direct effect accounting for 46.61% of the total effect. Additionally, moral injury can indirectly influence positive coping through the mediating effects of moral resilience and moral courage, as well as through the sequential mediation of both. The mediation effects account for 18.64%, 23.73% and 11.02% of the total effect, respectively.

**Conclusion:**

Moral injury is negatively correlated with positive coping style of nursing interns in China, and moral resilience and moral courage may buffer the negative effects of moral injury and improve the coping strategies.

## 1 Introduction

Clinical internships are a critical component of nursing education; however, nursing interns frequently face significant challenges and pressures throughout this period. Nursing interns were considered to be at high risk of moral harm, with 34.6% experiencing moral injury (Xie et al., 2019), and it’s at a severe level (Chen et al., 2020). Moral injury refers to the intense emotional distress that arises from experiences of violating moral or ethical principles (Zhang et al., 2023). As a highly significant stressor, if not addressed promptly, it may lead to more severe mental health problems. Firstly, due to the individual factors such as personal vulnerability, lack of clinical experience, and weak nurse–patient communication abilities, nursing interns frequently struggle to bridge the gap between theory and practice (Zhao et al., 2021). Nursing interns tend to experience low levels of self-compassion and high levels of self-criticism, which are common characteristic among individuals who suffer from moral injury (Zerach and Levi-Belz, 2021). Secondly, due to the lack of organizational factors such as experience guidance and mentor support, nursing students are more vulnerable to moral injury (Murray et al., 2018). Compared with experienced registered nurses, when nursing interns witness or fail to prevent these negligent acts that violate their deeply rooted moral beliefs, their moral code will be damaged, leaving deep emotional trauma, making them more vulnerable to moral injury (Hebert, 2020). More importantly, their limited professional competence in addressing potential moral injury incidents within clinical settings heightens vulnerability to moral injury (Murray et al., 2018). Insufficient clinical experience and immature judgment capacity impede intern nurses’ ability to navigate ethical challenges (Mbazo et al., 2025), potentially leading to the replication of unethical or unprofessional behaviors (Mewborn et al., 2023), or triggering psychological distress and diminished moral self-awareness.

Coping styles refer to the process by which individuals alleviate psychological burdens through cognitive and behavioral responses in stressful situations. When faced with stress, an individual’s coping mechanisms are automatically activated (Folkman et al., 1986; Guo et al., 2023). Based on the effectiveness of these responses, coping styles are typically categorized into positive coping (such as problem-solving and seeking social support) and negative coping (such as avoidance, denial, and neglect) (Jiao et al., 2023). In critical situations, positive coping can serve as a protective factor against mental health issues (Li and Chen, 2023), while negative coping is more likely to lead to feelings of guilt, shame, anger, and hopelessness (Xing et al., 2024). Research indicates that inadequate coping with moral injury among nursing interns may lead to shifts in values and professional attitudes, which increases the risk of burnout and even prompting some to consider leaving the nursing profession (Cheng et al., 2020). However, with the global aging population and the increasing burden of chronic diseases, nursing interns serve as a crucial reserve force for the nursing workforce (Cheng et al., 2020). The high attrition rate and professional burnout among nursing interns can undermine the quality of nursing care, impact patient safety, and even threaten the stability and sustainability of healthcare institutions (Sun et al., 2023).

Recently, the widespread application of positive psychology has introduced new approaches to enhancing nurses’ mental health. Study suggested that positive psychological resources can help nurses cope effectively with trauma, tragedy, threats, and significant work-related stress (Lee et al., 2020). Moreover, some scholars suggest that when individuals face stress, they can build resilience by identifying and mobilizing coping resources, with personal psychological resources such as moral resilience and moral courage playing a central role (Schäfer et al., 2023). Existing studies have demonstrated that these resources not only helped mitigate the negative effects of moral dilemmas and moral injury but also improved coping styles and prevent mental health issues (Kubitza et al., 2023; Spilg et al., 2022). Accordingly, this study aims to empirically examine the roles of moral resilience and moral courage in relation to moral injury and coping styles, providing theoretical support for enhancing nursing interns’ psychological well-being and coping styles.

### 1.1 Background

This study is based on the Transformed Model of Stress and Coping (TMSC) (Lazarus and Folkman, 1984; Lazarus and Alfert, 1964; Lazarus and Folkman, 1987) which includes three core elements: stressors, mediating factors, and stress responses. Lazarus’s theory posited a reciprocal relationship between stress and coping. On one hand, stress can stimulate or interfere with an individual’s coping mechanisms; on the other hand, coping styles can help reduce stress through psychological and physiological pathways. The revised Transformed Model of Stress and Coping model introduced new theoretical insights, highlighting the importance of positive emotions in the coping process, specifically noting that positive emotions aided in the restoration of physiological, psychological, and social coping resources (Folkman, 2008).

Rushton (2018) proposed a continuum of responses to imperiled integrity that reflects differences in intensity, sources, and consequences. The continuum begins with moral adversity and includes moral stress, moral distress, moral injury, moral decline, and moral apathy, outrage, or disengagement. This indicates that moral injury is a link between the previous and the next. Some scholars believe that the same variable can be interpreted in different ways, which can be regarded as either coping or the result after coping (Beeler et al., 2019; Jiang and Wang, 2022; Zychlinski et al., 2020). This is our understanding of moral injury. A systematic review described moral injury was predominantly positive associations with PTSD, depression, anxiety and suicidality; questionable associations with alcohol and other drug use; and potential associations in other health domains (e.g., physical health and treatment-seeking) (Hall et al., 2022). This suggests that moral injury can act as a stressor leading to different coping and outcomes. Based on this model, we hypothesize that nursing interns with lower levels of moral injury and higher levels of moral resilience and moral courage may achieve more positive coping outcomes.

Recently, cultivating moral resilience has become a central strategy for healthcare professionals to manage moral distress resulting from ethical dilemmas (Rushton, 2023). Moral resilience is defined as an individual’s ability to maintain or restore moral integrity when facing ethical challenges (Rushton et al., 2022). Research shows that moral resilience plays a sustaining and mediating role in the development of moral injury (Rushton et al., 2022). When the level of moral resilience is insufficient to withstand the impact of ethical dilemmas, individuals may experience moral injury. However, after the injury occurs, moral resilience helps alleviate the moral distress and severity of trauma in nurses, thereby assisting in maintaining their moral integrity (Berdida, 2023).

For healthcare professionals, the positive coping styles employed to address moral injury are also rooted in moral resilience. Certain coping styles can be reinforced through the cultivation of moral resilience (Faraco et al., 2022). Furthermore, research has indicated that moral resilience can strengthen the connection between self-coping styles and positive emotions, enabling nurses to approach ethical challenges more constructively and proactively, such as moral dilemmas or moral injury (Hossain and Clatty, 2021; Lathabhavan et al., 2023).

Moral courage is also regarded as one of the important means to alleviate ethical dilemmas. Moral courage refers to the ability of an individual to adhere to moral values and do the right thing, even when faced with known risks of adverse consequences (Dinndorf-Hogenson and Georgia, 2015). Research demonstrated a negative relationship between moral courage and moral injury, suggesting that moral courage indirectly reduces moral injury (Berdida, 2023). Nurses with high moral courage were more capable of maintaining ethical integrity when facing injustice or violations, thereby reducing the severity moral injury and alleviating associated psychological symptoms (Khoshmehr et al., 2020).

An individual’s coping styles in ethical dilemmas are also closely linked to their level of moral courage. Study indicated that nurses with high moral courage were more likely to employ positive coping style, while those with lower moral courage tended to experience negative coping emotions (Hong et al., 2023). Nurses with higher moral courage tend to seek solutions and make decisions that benefit patients and uphold ethical standards in challenging situations. This can help them alleviate the negative emotional distress caused by moral injury (Kelley et al., 2022).

Numerous studies have explored the relationship between moral resilience and moral courage. Study by Berdida (2023) had identified a significant positive correlation between moral resilience and moral courage. Further analysis by Abdollahi et al. (2021) identified predictors of resilience, revealing that moral courage accounts for 45% of the variance in moral resilience, which indicating a synergistic and mutually influential relationship.

Conceptual analysis reveals that although moral resilience and moral courage exhibit conceptual similarities, they possess distinct definitions and should be regarded as independent constructs (Rushton, 2024). Moral resilience primarily denotes a psychological trait enabling individuals to restore and maintain internal equilibrium after ethical conflicts, whereas moral courage specifically refers to the immediate behavioral trait of taking fearless actions to uphold moral principles during such conflicts. From a dimensional perspective, moral resilience sustains adherence to personal values and convictions, thereby facilitating morally aligned actions—a hallmark of integrity. Crucially, preserving personal integrity constitutes a necessary condition for exercising moral courage in ethical dilemmas (Young and Rushton, 2017). This framework elucidates how moral resilience influences moral courage behaviors. Empirical evidence further corroborates that when individuals confront conflicts threatening moral integrity, those with higher moral resilience demonstrate stronger tendencies to uphold ethical standards and initiate integrity-preserving actions—an observable manifestation of moral courage (Monteverde, 2016).

While existing researches addresses moral injury, coping styles, moral courage, and moral resilience in nursing interns, few studies explore the relationships and mechanisms connecting these four variables. Furthermore, most recent research focuses on the psychological and pathological consequences of moral injury, with limited attention to its specific effects on individual behavior. Particularly within the Chinese context, the impact of moral injury on nursing interns’ coping styles remains unclear, highlighting the need for further research.

### 1.2 Aims

The objectives of this research were to explore the relationship between moral injury, moral resilience, moral courage and positive coping for nursing interns in clinical practice, and then to explore the direct and indirect pathways between moral injury and positive coping.

### 1.3 Hypothetical model

**Figure 1:**
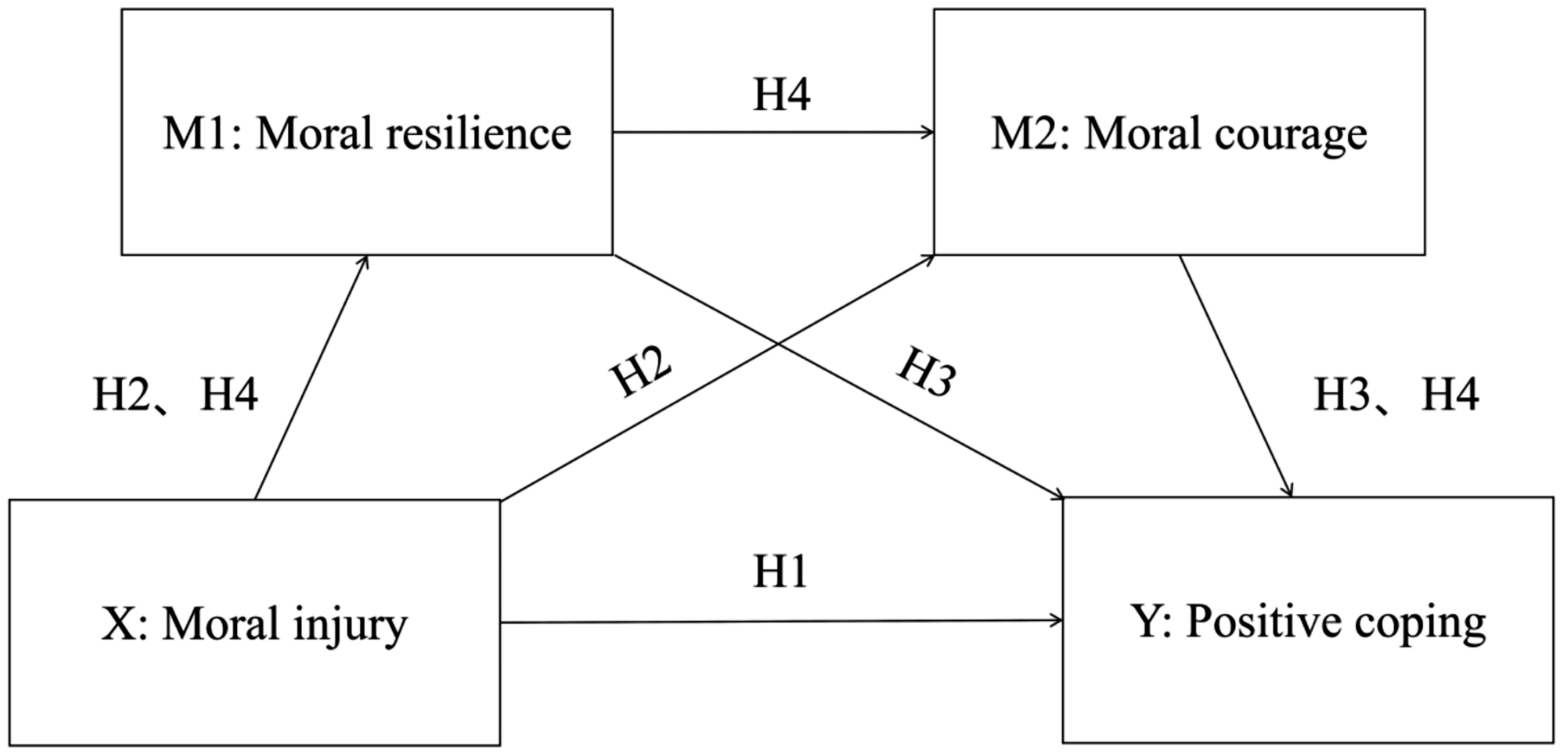
Hypothesized relationship among moral injury, moral resilience, moral courage, and positive coping style.

In this study, a hypothesized model (Figure 1) was established based on the Transformed Model of Stress and Coping Model to test four hypotheses.

*H1*: Moral injury negatively influences positive coping style.

*H2*: Moral injury negatively influences moral resilience and moral courage.

*H3*: Moral resilience and moral courage positively influence positive coping style.

*H4*: Moral resilience and moral courage form a chain mediation from moral injury to positive coping style.

## 2 Methods

### 2.1 Study design

This is a multicenter and self-reported cross-sectional study that followed the STROBE guidelines. Sampling and data collection have been carried out completely online.

### 2.2 Participants and setting

From February 1 to April 15, 2024, this study recruited nursing interns using a multi-stage sampling method in 4 provincial capital cities in Southwest China (Kunming, Guiyang, Chengdu, and Chongqing). One college and/or one university (either junior college or undergraduate) was selected in each city, with 3 classes sampled from each selected institution. A total of 6 institutions and 18 classes (13 junior college classes and 5 undergraduate classes) were finally included in this study.

Participants were approached if they met the inclusion criteria: (1) full-time nursing interns; (2) at least 4 weeks in clinical practice; (3) informed consent and voluntary participation. Exclusion Criteria: (1) terminating the internship for any reason; (2) taking sick leave or personal leave during the investigation.

An online calculator developed by Schoemann et al. (2017) determined the desired sample size (website: https://schoemanna.shinyapps.io/mc_power_med/). We chose the ‘two serial mediators’ model and the ‘Set Power, Vary N’ option, selected 5,000 replications and 95% confidence intervals, and calculated the required sample size using Monte Carlo power analysis. The entered correlation matrix was chosen according to recommendations from previous research (Schoemann et al., 2017). This indicated that 519 individuals were required to ensure that the statistical power would be at least 80% for testing our hypotheses. A total of 1,122 participants were finally included in this study.

### 2.3 Instruments

The participants’ social demographic profiles include sex, age, place of residence, single child, education level, internship stage, whether nurse is the first choice of the college, professional score, professional recognition, whether to practice nursing after graduation, and have you studied nursing ethics.

The symptoms of moral injury in nursing interns was measured by *the 10-item Moral Injury Symptom Scale: Healthcare Professionals Version (MISS-HP)*, developed by Mantri et al. (2020). The Chinese version of Moral Injury Symptom Scale: Healthcare Professionals Version was translated by Zhizhong et al. (2020). These 10 items were answered using a visual analog scale with options ranging from 1 (strongly disagree) to 10 (strongly disagree). To reduce response bias, six of the items were negative and four were positive. Higher scores indicate further moral injury. The Cronbach’s *α* coefficient of the Chinese version of 10-item Moral Injury Symptom Scale: Healthcare Professionals Version was 0.71 and was 0.751 in this study.

Nursing interns’ coping style was measured by *the 20-item Simplified Coping Style Questionnaire (SCSQ)*, developed by Xie (1998). The scale involves the different attitudes and measures that people often take in daily life, and can reflect the characteristics of different coping styles and the relationship with mental health. According to different coping styles, it was divided into two dimensions: positive coping style and negative coping style, with a total of 20 items. Each item was rated on a four-point Likert scale (3 = do often to 0 = do not). This study assesses the level of positive coping among nursing interns using the positive coping dimension, which scores range from 0 to 36. Higher scores indicate a greater level of positive coping. The Cronbach’s *α* coefficient of the Chinese version of the 20-item Simplified Coping Style Questionnaire was 0.911, and 0.89 for the positive coping dimension, while this study found values of 0.912 and 0.929.

Nursing interns’ moral resilience was measured by *the 17-item Rushton Moral Resilience Scale (RMRS)*, developed by Heinze et al. (2021). Tian et al. (2024) translated the original RMRS into Chinese and performed cross-cultural adaption based on Chinese clinical professional nurses. The Chinese version of the 17-item Rushton Moral Resilience Scale were categorized into 3 dimensions, including the ability to respond flexibly to moral adversity, relational moral soundness, and moral efficacy. Each item was rated on a four-point Likert method (1 = disagree to 4 = agree). In this scale, a total of 11 items are the negative questions and should be reversely scored. The total score for each dimension is the mean of all items belonging to that dimension, and the total score for this scale is the mean of all 17 items. Higher scores indicate greater moral resilience. The Cronbach’s *α* coefficient of the Chinese version of the 17-item Rushton Moral Resilience Scale was 0.922. This study showed an overall alpha of 0.884.

Nursing interns’ moral courage was measured by *the 21-item Nurses’ Moral Courage Scale (NMCS)*, developed by Numminen et al. (2019). Siyao et al. (2019) translated this scale into the Chinese version used in this study. The scale consists of four dimensions and 21 items, including moral integrity, commitment to good care for patients, compassion and true presence with patients and moral responsibility. Each item was rated on a five-point Likert method (1 = completely inconsistent with me to 5 = completely consistent with me). The higher the score, the higher the level of moral courage of nursing interns. The Cronbach’s α coefficient of the Chinese version of the 21-item Nurses’ Moral Courage Scale was 0.905. This study showed an overall alpha of 0.966.

### 2.4 Data collection

The anonymous questionnaire survey was carried out online through the “Questionnaire Star” platform^1^, relying on WeChat (the largest Chinese social media platform). The class counselors of the nursing schools sent the electronic questionnaire QR Code generated by “Questionnaire Star” to the Wechat Group where the clinical practice nursing interns were located, and explained the informed consent and exclusion criteria uniformly, which were filled out by the voluntary scanning QR Code of nursing interns. The questionnaire was administered in Chinese with uniform instructions to explain the purpose, significance and filling method of this survey. To ensure data quality, the following validation protocols were implemented via Questionnaire Star: Single submission per WeChat ID to prevent duplicates, submission permitted only after all required items were answered and responses with completion time <3 min were invalidated. Study data accessed via Questionnaire Star’s secure login credentials were downloaded in Excel format and encrypted to ensure participant privacy. A total of 1,164 questionnaires were collected, and 42 including same score and across all items were eliminated. Finally 1,122 questionnaires were suitable for analysis after data cleaning, with an effective recovery rate of 96.39%.

### 2.5 Data analysis

Data were processed using SPSS 23.0 and the PROCESS 4.1 macro. All collected questionnaires were screened, and data from valid responses were imported into SPSS 23.0 for summarization and organization. Internal consistency of each questionnaire was assessed via reliability analysis. Descriptive statistics were used to analyze participants’ sociodemographic characteristics, with results presented in terms of mean, standard deviation, and percentage. Pearson correlation analysis was conducted to explore relationships between variables. Finally, Hayes’ PROCESS macro (Model 6) was employed to test the hypothesized model (Hayes, 2013).

### 2.6 Ethics approval

The study was reviewed by the Research Ethics Committee of the our hospital, which granted permission to conduct the study (number: K2023-045). The study followed the ethical guidelines, protocol, and regulations stated in the Declaration of Helsinki. Informed consent was obtained from all participants.

## 3 Results

### 3.1 Common method bias

Regarding the actual survey procedure, all questionnaires were filled in anonymously, and regular integrals and inversion integrals were used to control potential method bias. Harman’s single factor test was used for exploratory factor analysis to test common method bias. The results indicated that there were 8 factors with eigenvalues greater than 1, among which the first factor explains 28.45% of the variance, which was below the critical threshold of 40%. This demonstrates the absence of common method bias in this study, making it suitable for further analysis.

### 3.2 Participants’ demographic profiles

The participants’ demographic profiles are presented in Table 1. A total of 1,122 participants were included in the study. There were 970(86.45%) female and 152(13.55%) male. The mean (SD) age was 20.21 ± 1.70. There were 918(81.82%) studied for Junior college nursing interns (3-year program) and 204(18.18%) for undergraduate nursing interns (4-year program). There were 587(52.32%) in the early stages (5–12 weeks) of internship stage, 73(6.51%) in the middle stage (13–28 weeks) and 462(41.18%) in the later stage (29–42 weeks). There were 853(76.02%) had studied nursing ethics.

**Table 1:** General information of nursing interns (*N* = 1,122).

Variable	*n*(%)
Individual factors	
Sex	
Male	152(13.55)
Female	970(86.45)
Age (years)	20.21 ± 1.70
Place of family residence	
Cities and towns	355(31.64)
Rural area	767(68.36)
Single child	
Yes	244(21.75)
No	878(78.25)
Education level	
Junior college nursing interns (3-year program)	918(81.82)
Undergraduate nursing interns (4-year program)	204(18.18)
Internship stage	
Early stage (5–12 weeks)	587(52.32)
Middle stage (13–28 weeks)	73(6.51)
Later stage (29–42 weeks)	462(41.17)
Professional learning factors	
Whether nurse is the first choice of the college	
Yes	800(71.30)
No	322(28.70)
Professional score	
Very good	93(8.29)
Good	390(34.76)
Medium	578(51.52)
Poor	50(4.45)
Very poor	11(0.98)
Professional recognition	
Very low	24(2.14)
Low	44(3.92)
Medium	529(47.15)
High	404(36.01)
Very high	121(10.78)
Whether to practice nursing after graduation	
Yes	889(79.23)
No	233(20.77)
Have you studied nursing ethics	
Yes	853(76.02)
No	269(23.98)

### 3.3 Correlations between moral injury, moral resilience, moral courage and positive coping style

It can be noted that the mean scores of moral injury, moral resilience, moral courage, positive coping scores and negative coping scores were 40.07 ± 11.22, 46.54 ± 5.91, 72.67 ± 15.75, 23.56 ± 7.13 and 10.82 ± 5.02, respectively (Table 2).

**Table 2:** The score of moral injury, moral resilience, moral courage and coping style of nursing interns (*N* = 1,122).

Variable	Item	Mean score	Mean score of each item
Total moral injury	10	40.07 ± 11.22	4.80 ± 1.44
Total moral resilience	17	46.54 ± 5.91	2.74 ± 0.35
Ability to respond flexibly to moral adversity	5	12.89 ± 3.32	2.58 ± 0.66
Relational moral soundness	6	15.61 ± 3.52	2.60 ± 0.59
Moral efficacy	6	18.04 ± 3.17	3.01 ± 0.53
Total moral courage	21	72.67 ± 15.75	3.46 ± 0.75
Moral integrity	7	23.74 ± 5.43	3.39 ± 0.78
Commitment to good care	5	17.23 ± 4.09	3.45 ± 0.82
Compassion and true presence	5	17.37 ± 4.09	3.47 ± 0.82
Moral responsibility	4	14.34 ± 3.36	3.59 ± 0.84
Positive coping scores	12	23.56 ± 7.13	3.88 ± 1.43
Negative coping scores	8	10.82 ± 5.02	1.35 ± 0.63

The correlation matrix depicted that moral injury was negatively associated with moral resilience (*r* = −0.463, *P* < 0.001), moral courage (*r* = −0.226, *P* < 0.001) and positive coping scores (*r* = −0.235, *P* < 0.001). Moral resilience was positively correlated to moral courage (*r* = 0.184, *P* < 0.001) and positive coping scores (*r* = 0.196, *P* < 0.001). Additionally, moral courage was positively associated with positive coping scores (*r* = 0.515, *P* < 0.001) (Table 3).

**Table 3:** Correlation analysis of moral injury, moral resilience, moral courage and positive coping (*N* = 1,122).

Variable	Total moral injury	Total moral resilience	Total moral courage	Positive coping scores
Total moral injury	1			
Total moral resilience	−0.463**	1		
Total moral courage	−0.226**	0.184**	1	
Positive coping scores	−0.235**	0.196**	0.515**	1

**P < 0.001.

### 3.4 Mediating effects of moral resilience and moral courage on the relationship between moral injury and positive coping style

**Figure 2:**
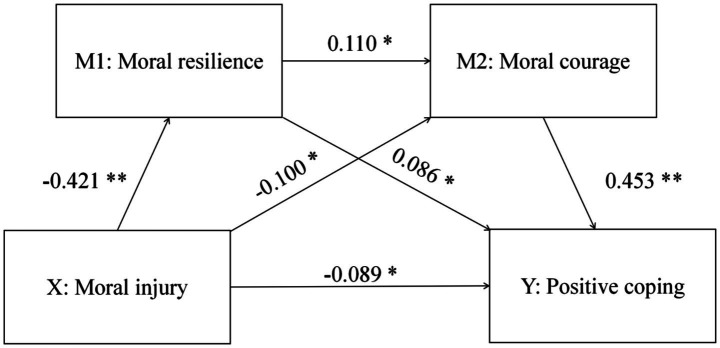
Confounder-adjusted mediation model. *P < 0.01, **P < 0.001.

First, a multicollinearity test was conducted for all regression equations. The results showed that all predictor variables met the criteria of variance inflation factor (VIF) < 3 and minimum tolerance > 0.3, indicating no severe multicollinearity in this study. Moral injury was specified as the independent variable, with positive coping as the dependent variable. Moral resilience and moral courage were included as sequential mediators. Covariates comprised sex, age, place of residence, single child, education level, internship stage, whether nurse is the first choice of the college, professional score, professional recognition, whether to practice nursing after graduation, and have you studied nursing ethics. Model 6 in the PROCESS macro was used to further examine the sequential mediation effect (Table 4 and Figure 2). Regression analysis showed that the combined predictive effect of moral injury, moral resilience, and moral courage scores on positive coping scores was 31.60%.

**Table 4:** Regression analysis of moral resilience and moral courage between moral injury and positive coping (*N* = 1,122).

Model	Variable	Adjust *R*^2^	*F*	Beta	SE	*t*	95%CI	
							LLCI	ULCI
Outcome: moral resilience	Constant	0.260	32.484	—	2.615	20.815**	49.298	59.559
	Moral injury			−0.421	0.014	−15.471**	−0.250	−0.194
Outcome: moral courage	Constant	0.105	9.951	—	9.047	5.360**	30.739	66.243
	Moral injury			−0.100	0.046	−3.016*	−0.231	−0.049
	Moral resilience			0.110	0.088	3.319*	0.120	0.465
Outcome: Positive coping	Constant	0.316	36.473	—	3.510	−0.093**	−7.213	6.561
	Moral injury			−0.089	0.018	−3.067*	−0.090	−0.020
	Moral resilience			0.086	0.034	2.960*	0.034	0.167
	Moral courage			0.453	0.012	17.255**	0.176	0.221

*P < 0.01, **P < 0.001. All models have control for the variables: sex, age, place of residence, single child, education level, internship stage, whether nurse is the first choice of the college, professional score, professional recognition, whether to practice nursing after graduation, and have you studied nursing ethics.

Beta, standardized coefficients; CI, confidence interval; LLCI, lower limit confidence interval; SE, standard error; ULCI, upper limit confidence interval.

The results indicated a significant negative correlation between moral injury and each of moral resilience, moral courage, and positive coping, thereby supporting Hypotheses 1 and 2. Additionally, moral resilience and moral courage were significantly positively correlated with positive coping, supporting Hypothesis 3. After adding covariates, moral resilience and moral courage significantly moderated the influence of moral injury on positive coping. Furthermore, moral resilience directly influenced moral courage, supporting Hypothesis 4.

Using the Bootstrap method with 5,000 resamples, we calculated 95% confidence intervals for the model’s total effect, direct effect, and indirect effect. The 95% confidence intervals for each effect did not contain zero, indicating that moral resilience and moral courage each served as mediators between moral injury and positive coping (with effect sizes of 0.022 and 0.028, respectively) and functioned as a sequential mediator (effect size 0.013). The total effect of moral injury on coping styles 0.118, with a direct effect of 0.055 and an indirect effect of 0.063. The mediating effect of moral resilience and moral courage accounted for 53.39% of the total effect, as shown in Table 5.

**Table 5:** The direct and indirect effect of moral injury on positive coping (*N* = 1,122).

Effect	Effect value	Bootstrap SE	LLCI	ULCI	VAF
Total effect	−0.118	0.018	−0.153	−0.082	100%
Direct Effect	−0.055	0.018	−0.090	−0.020	46.61%
Indirect Effect	−0.063	0.013	−0.088	−0.038	53.39%
Moral injury – moral resilience – positive coping	−0.022	0.008	−0.038	−0.007	18.64%
Moral injury – moral courage – positive coping	−0.028	0.010	−0.049	−0.007	23.73%
Moral injury – moral resilience – moral courage – positive coping	−0.013	0.005	−0.022	−0.004	11.02%

LLCI, lower limit confidence interval; ULCI, upper limit confidence interval; VAF, the variance accounted for.

## 4 Discussion

Our research findings fully confirm our hypothesized model. We found that moral injury can influence coping strategies both directly and indirectly, as well as impact positive coping through the sequential mediation of moral resilience and moral courage. These findings enhance our understanding of how moral injury affects coping styles and provide theoretical support for guiding nursing students in making sound decisions and actions.

Our findings showed that the severity of moral injury among nursing interns in China was at a moderately low level, with scores significantly below those of Chinese nurses (46.3 ± 12.2) as reported by Zhizhong et al. (2020), and the average item score of (7.16 ± 0.41) reported by Berdida and Grande (2023) for Filipino nurses. This disparity may be attributed to differences in data collection periods and participant demographics. The studies by Zhizhong et al. (2020) and Berdida and Grande (2023) took place during the COVID-19 pandemic, a period marked by high rates of life-threatening infections and critical shortages in medical resources, leaving clinical nurses unable to provide adequate patient care. This situation forced nurses to make difficult decisions, such as allocating ventilators or beds in intensive care units. These experiences left frontline nurses feeling helpless, ashamed, and guilty, which contributed to severe moral injury (Coimbra et al., 2023). The positive coping dimension score for Chinese nursing interns was at a moderately high level, consistent with the findings of Wan et al. (2023) on Chinese undergraduate nursing students, demonstrating that our participants generally displayed a more active approach and problem-solving ability when coping with stress. This tendency may be related to the fact that 75% of the nursing interns had received training in medical ethics and moral topics during their studies.

In line with the TMSC model and prior research, our findings confirmed that moral injury had a direct negative impact on positive coping style. This finding had not previously been reported in studies on nursing interns in China. This aligns with the theoretical proposition put forward by Xie et al. (2019). Specifically, Xie and their associates directly established a connection between stressors and coping, arguing that stressors serve as the initiating factor for an individual’s coping process. This represents a derivative application of TMSC theory. Moral injury is a unique and intense source of psychological stress, representing a relatively new area within the field of psychological trauma. Whether a stressor (moral injury) triggers a stress response (mental health problem) depends on the nursing interns’ cognitive appraisal of moral injury, for example, whether he or she has enough resources to deal with moral injury (Lazarus and Folkman, 1987). Different nursing interns have different cognitive appraisal of moral injury and different coping style (Zhang et al., 2023). Positive coping style have a beneficial effect on lifestyle (Jin et al., 2024). Medical students who preferred positive coping style can use purposeful, flexible coping methods to address challenges, mitigating stressors like moral injury and supporting their physical and mental well-being (Cheng et al., 2020). However, in the face of moral injury, nursing interns may have difficulty maintaining clear judgment or implementing positive coping due to emotional burden, which can increase emotional distress and occupational stress. By assessing coping resources, it helps nursing interns to implement positive coping styles and more effectively manage mental health problems caused by moral injury, thus promoting better understanding and coping with similar challenges in the future. This finding underscores the importance and necessity, in the nursing field, of early identification of stressors and the adoption of positive coping styles to alleviate moral injury in the face of frequent professional ethical challenges.

The mediation analysis results showed that moral injury exerts a significant indirect influence on positive coping style through moral resilience and moral courage. Researchers suggested that resilience and courage were not merely intrinsic psychological traits but also psychological resources that can be effectively mobilized when nursing interns encounter moral injury (Berdida, 2023; Hong et al., 2023). These resources can enhance nursing interns’ positive emotions and encourage them to adopt more constructive and proactive approaches to problem-solving, thereby mitigating the negative impact of moral injury on long-term psychological well-being (Berdida, 2023; Hong et al., 2023).

First, moral resilience plays a significant mediating role in the relationship between moral injury and positive coping style. Nurses with higher moral resilience levels are more likely to adopt positive coping style in response to moral injury, a finding consistent with previous researches (Rushton et al., 2022; Talebian et al., 2022). Studies indicated that moral resilience helps nurses maintain psychological stability in stressful environments, countering moral injury’s negative effects. This mechanism is especially valuable as moral dilemmas intensify, with nurses frequently relying on it to remain engaged in their work positively and consistently (Rushton et al., 2022; Talebian et al., 2022). For example, during the COVID-19 pandemic, nurses cultivated their moral resilience through mindfulness practices and by strengthening their parasympathetic nervous systems via exercises such as deep breathing. These practices helped them respond positively to stress (Hossain and Clatty, 2021).

Second, moral courage mediates the relationship between moral injury and positive coping style, a finding consistent with existing literature. According to Mewborn’s finding (Mewborn et al., 2023), moral courage played a crucial role in reinforcing moral decision-making and behavioral performance, enabling clinical nurse to act under moral pressure rather than feeling helpless. Similarly, Abdollahi et al. (2021) suggested that nurses with higher levels of moral courage, possessing characteristics such as the ability to endure threats, maintain moral objectives, and balance multiple values, have a realistic understanding of their capabilities. This understanding allows them to remain adaptable and adopt positive coping measures when faced with threats and stressors.

Finally, consistent with Hypothesis 4, moral courage and moral resilience demonstrate a serial mediation effect on the relationship between moral injury and positive coping style, accounting for 53.39% of the total effect. This study contributes by highlighting the facilitative role of moral resilience in fostering moral courage, suggesting that these two factors interact to improve Chinese nursing interns’ ability to cope positively with moral injury. Similar findings were observed by Berdida (2023) who found that moral resilience positively influenced moral courage. Including moral resilience and moral courage as mediators may reduce the impact of moral dilemmas and moral injury. When clinical nurses encounter moral dilemmas, moral resilience enables them to handle stressors from management practices (Bruggmann et al., 2023); those with high psychological resilience can develop sound judgment and understanding, thereby fostering confident moral courage behaviors to navigate the challenges (Khoshmehr et al., 2020). Further research by Yang et al. (2023) demonstrated a strong positive correlation between resilience and moral courage, with a direct effect reaching 85%. Moral courage emphasizes action, whereas resilience focuses on psychological endurance; together, these factors synergistically support mental health and moral integrity (Oser and Reichenbach, 2005).

### 4.1 Limitations

Despite the valuable insights gained from this study, several limitations remain. First, data collection relied primarily on self-reported measures, which may be influenced by social desirability bias, leading nursing interns to report more idealized coping styles rather than actual emotional responses. Future studies could incorporate objective measures, like behavioral observations and third-party assessments, to reduce self-reporting bias. Second, while this study examined the mediating roles of moral resilience and moral courage, other potential mediators—such as social support, work environment, and personal beliefs—were not fully explored. Future research could explore the mediating or moderating effects of these factors on the relationship between moral injury and coping styles for a more comprehensive understanding of coping mechanisms in moral injury. Last, this study has geographical limitations. The sample only includes nursing interns from Southwest China and does not cover other regions. Due to the significant differences in nursing education systems, internship environments, and medical policies across different provinces, the promotion of the research results to other regions of china may be restricted. Therefore, caution should be exercised when generalizing the results to the whole country. Future studies should include samples from more regions to enhance the external validity of the results.

## 5 Conclusion

This study, using a multiple mediation model, confirmed the direct impact of moral injury on positive coping style, and the indirect mediating roles of moral resilience and moral courage. Moral injury may reduce nursing interns’ willingness and ability to adopt positive coping style when facing moral challenges. However, moral resilience and moral courage can help mitigate the negative effects of moral injury and influence behavioral choices, making it more likely that nursing interns will adopt constructive actions in the face of moral adversity. This study offers a targeted theoretical model and practical recommendations for addressing moral injury among nursing interns, advancing the field of moral psychology within nursing practice.

## 6 Relevance for clinical practice

Nursing interns inevitably face moral injury during their internships. The findings of our study provides clear guidance strategies for nursing managers and educators: by enhancing ethical resilience, moral courage, and coping skills, the negative psychological effects of moral injury on nursing students can be alleviated and it can promote the adoption of positive coping methods. Specifically, on the one hand, schools can introduce nursing ethics courses in advance. Scenario-based simulation courses on moral dilemmas and moral injury can be added before nursing students begin their clinical internships, enabling them to more effectively manage emotions and stress in complex ethical dilemmas. On the other hand, medical institutions need to create an organizational atmosphere of nursing ethics and establish a nursing support system. For example, establishing a supportive work environment to enhance moral resilience and courage through regular psychological support, professional ethics training and ethical discussion, to promote the ability of nurses to adopt positive coping styles when facing challenges (Hossain and Clatty, 2021). These approaches not only contributes to the professional development of nursing staff, but also improves the quality of nursing care provided.

## Data Availability

The original contributions presented in the study are included in the article/supplementary material, further inquiries can be directed to the corresponding author.

## Footnotes

^1^ https://www.wjx.cn/vm/YDN6MNO.aspx
